# Experimental effects of paracetamol overdose on the development of forensic insects

**DOI:** 10.1007/s12024-025-01168-4

**Published:** 2026-02-08

**Authors:** Nathania Esther Munsami, Samson Mukaratirwa, Danisile Tembe

**Affiliations:** 1 School of Agriculture and Science, University of KwaZulu-Natal, Westville Campus, Private Bag X54001, Durban, 4000 South Africa; 2 One Health Center for Zoonoses and Tropical Veterinary Medicine, Ross University School of Veterinary Medicine, Basseterre, Saint Kitts and Nevis

**Keywords:** Postmortem interval, Forensic entomotoxicology, Paracetamol, Insect development, Morphology, Morphometry

## Abstract

In forensic entomology, postmortem interval (PMI) estimation relies on the age, development, and succession patterns of insects on decomposing remains; however, the presence of drugs in the body can disrupt insect development and compromise the accuracy of these estimates. Paracetamol, a commonly used over-the-counter drug in Southern Africa, is often implicated in both intentional and accidental overdose cases. This study investigates the effects of paracetamol on insect development using spiked pig carcasses as experimental models to simulate human overdose. Three pigs received paracetamol at ascending doses 75 mg/kg (toxic), 150 mg/kg (lethal), and 300 mg/kg (double lethal), and the fourth pig served as an untreated control. The experimental carcasses were placed in separate metal cages in a natural outdoor environment during the autumn season, and insect samples were collected daily over a 32-day period. Observations and morphological measurements of the length, width, and weight were recorded for selected dipteran and coleopteran species of forensic value. Results showed that *Chrysomya putoria* and *Chrysomya megacephala* exhibited an increased larval length, width, and weight at double lethal doses. *Lucilia sericata* showed significant reductions in weight at all paracetamol doses (*p* = 0.012, 0.001 and 0.001) in comparison to the control, while *Chrysomya albiceps* and *Thanatophilus micans* were not affected by the drug. Furthermore, results showed that the mortality rates of newly emerged *Ch. putoria*, *Ch. megacephala*, *Ch. albiceps* and *L. sericata* were higher in paracetamol-treated groups compared to those observed from the control group, with the rates of 22.2% at the toxic dose, 35.0% at the lethal dose, 45.0% at the double lethal dose, and 11.1% in the control group. Morphological abnormalities were observed in newly emerged adults of *Ch. putoria* and *L. sericata* that showed progressive wing deformities at the toxic and lethal doses and discoloration at the double lethal dose. *Ch. albiceps* exhibited no observable deformities across all doses. These findings show that paracetamol disrupts critical developmental processes in certain species in a dose-dependent manner, highlighting the need to study its effects on other forensically important insects. Moreover, these findings contribute towards enhancing PMI precision and advancing forensic entomotoxicology in the region by generating local baseline data for KwaZulu Natal province, South Africa.

## Introduction

Drug poisoning due to accidental or intentional drug overdose is a significant public health concern globally [1]. According to the World Health Organization in 2023, unintentional drug poisoning caused approximately 69,000 deaths annually, while intentional poisoning accounted for 188,000 deaths. In South Africa, a survey conducted by the National and Provincial Departments of Health reported that unintentional deaths comprised 20% of poisoning cases, with suicides constituting 13% between 2020 and 2021 [2]. Among the substances implicated in these poisoning incidents, paracetamol is the leading cause of intentional and accidental drug poisoning accounting for 41% of recorded death cases [1]. Paracetamol is often subject to misuse, largely attributed to its over-the-counter accessibility at local pharmacies/drug stores and is commonly used in the treatment of everyday ailments such as pain and fever.

In cases of drug overdose deaths, forensic investigators analyse body fluids (i.e., blood or urine), tissues and organs, to determine the presence of drugs or poison and the post-mortem interval (PMI) of a deceased [3, 4]. However, as decomposition progresses, autolytic and putrefactive processes degrade these body organs causing them to be undetectable or unsuitable for toxicological analysis [5, 6]. Studies have also shown that the reliability of biological samples for toxicological analysis reduces after 72 h, highlighting the need for alternative methods to accurately establish the time and cause of death [7]. In these cases, toxicological analysis from insects serves as a valuable alternative to determine indirectly the presence of drugs or toxicants from cadavers [6, 8, 9]. This field of study is known as forensic entomotoxicology [10]. Forensic entomotoxicology examines the presence of drugs and poisons in insects feeding on intoxicated carcasses and their effects on insect development or metamorphosis. Thus, providing valuable information for determining the cause of death [6, 11, 12].

Several studies have demonstrated that insects are not only limited to drug detection, but they are also used in PMI estimation which is estimated by analysing the age of the oldest insects found on a carcass, insect development and succession pattern [13]. In such cases, the knowledge of the life cycle of carrion associated insect plays a major role in PMI estimation [14]. However, insect development is influenced by humidity [6, 15], temperature [16, 17], altitude [6, 18], food availability [19, 20] and the presence of drugs and/or poisons in the cadavers [6, 21]. Additionally, it has been reported that the presence of drugs and toxins may accelerate or delay the growth rate [22] and alter the morphology of carrion feeding insects [12]. Consequently, these effects may result to an overestimation or underestimation of PMI during forensic investigation.

Much of the existing research has focused on a limited range of common drugs (i.e., morphine, cocaine, amphetamines, benzodiazepines) and on selected blowfly species [13]. According to Soni et al. [23], different drugs may have similar and/or different effects on the developmental stages of carrion feeding insects. Therefore, the pharmacological effects of one drug cannot be generalized for all drugs and all insect species. It is, therefore, important to understand the effect of different drugs and doses on the development of different insect species which feed on the carcass to correctly estimate PMI using entomological evidence [6, 21] and advance the application of forensic entomotoxicology in medicolegal investigations in different regions [13]. Given the high incidence of suicide by paracetamol in this region, it is vital to understand its effects on insects used in forensic investigations. This study therefore experimentally evaluated how different paracetamol doses affect the growth and development of insects feeding on intoxicated pig carcasses, with the aim of improving the accuracy of PMI estimation in KwaZulu-Natal, South Africa.

## Materials and methods

### Experimental animals

Four healthy pigs (*Sus scrofa domesticus*) with an average live weight of 35 kg were purchased from the Misty Mount Farm in Pietermaritzburg, KwaZulu-Natal province, and transported to the Biomedical Resources Unit (BRU), University of KwaZulu-Natal for the experiments. Upon arrival at the BRU, the pigs were weighed, labelled, and housed in a designated animal room for three days.

### Administration of paracetamol and euthanasia of experimental animals

Paracetamol tablets were purchased from a local pharmacy (Dischem pharmacy, South Africa). The drug cocktail was prepared by crushing 36 tablets (500 mg/tablet) to a powder form. Paracetamol powder was then diluted in 15 mL of sterile water, which served as the injectable diluent. To ensure a uniform distribution of the drug, the mixture was vortexed for 30 s. Prior to drug administration, the pigs were sedated using a combination of 2.21 mg medetomidine, 7 mg midazolam, and 7 mg butorphanol. Thereafter, three pigs were each administered paracetamol doses of 75 mg/kg (toxic), 150 mg/kg (lethal), and 300 mg/kg (double lethal) [24], via intramuscular injection. These dosages were adopted from those commonly reported in paracetamol suicide and accidental overdose cases [25–31]. Therapeutic doses were not applicable because individuals exposed to such levels do not succumb to paracetamol, and consequently, their tissues would contain only trace or metabolized amounts of the drug, unlikely to meaningfully affect post-mortem insect development. One pig served as a control and was not treated with paracetamol.

After 15–30 mins post-injection, 5 mL of blood was collected from the jugular vein of each pig using EDTA blood collection tubes to confirm the presence of paracetamol following drug administration and stored in -20 °C freezer. An hour post-injection all four pigs were sacrificed with 10 mL barbiturates (Euthanaze). Once the pigs were confirmed dead each pig carcass was placed in a separate labelled plastic bag and sealed with zip ties to prevent contamination. The carcasses were then transported to the experimental site within 1.5 h post-mortem to preserve biological integrity and ensure reliable toxicological analysis. The decomposition experiments were conducted at the University of KwaZulu-Natal’s Ukulinga Research Farm (30°24’S, 29°24’E). Upon arrival, the carcasses were placed in individual metal cages (100 × 50 × 50 cm) covered with wire mesh and positioned 50 m apart to prevent insect cross-contamination between treatment groups [32, 33]. The cages allowed the contact between the decomposing carcasses and the soil.

### Insect sampling, observation of carcasses and data collection

The study was conducted from 25 April to 27 May 2024, spanning 32 days of decomposition under autumnal conditions in Pietermaritzburg, South Africa. The day in which the animals were sacrificed and placed in metallic cages was designated as day 0. The sampling commenced and continued daily until the carcasses were dry with little to no active insects on the carcasses. Data and sample collection were done daily from day 0 to day 31 between 09:00 am to 14:00 pm. The ambient temperature, carcass body temperature, and soil temperature from which the carcasses were decomposing were recorded daily using a HW-F7 Simzo Digital infrared thermometer (Dongguan Simzo Electronic Technology Co., Ltd). Furthermore, carcasses were observed daily for post-mortem changes associated with different stages of decomposition [33, 34].

Immature insects were collected from various body parts of the carcasses, including the mouth, ears, abdomen, underneath the body, and surrounding soil, using sterilized forceps. From day 15 onwards, newly emerged adult insects were captured using two redtop bait fly traps (40 cm (length) x 30 cm (width) x 8 cm (height)) with non-poisonous protein meal bait placed 5 cm from the carcass suspended around the cages as well as two rectangular pitfall traps (7 cm(L) × 12 cm (W) × 8 cm (H)) placed 5–10 cm from the carcass and by hand-picking with forceps throughout the remainder of the decomposition process.

On each sampling day, 10–15 larvae were collected per carcass with samples consistently taken from the same anatomical regions across all stages of decomposition. Insect activities and their developmental patterns including oviposition day, emergence of first, second and third instars, and pupal stages were observed and recorded respectively. Additionally, morphological abnormalities on insects collected were also recorded. Larvae samples were sacrificed by immersing in 80 °C water for 30 s, then dried and stored in clean Eppendorf tubes at -20 °C. Newly emerged adults were sacrificed by ethyl acetate and preserved in a freezer (-20 °C).

### Measurement of body length, weight and width of collected insects

Ten larval specimens were randomly selected from the collections and individually placed into sterilized petri dishes [35]. Each larva was rinsed twice with 500 µL of distilled water to remove excess contaminants and then gently dried with a paper towel [35]. Prior to measuring the body length, weight, and width of the collected insect, individual samples were identified morphologically using identification keys [36–40]. Of all insect species that visited the carcasses, the study specifically focused on four dipterans species: *Chrysomya putoria*, *Chrysomya megacephala*, *Chrysomya albiceps*, and *Lucilia sericata* and one dominant coleopteran species, *Thanatophilus micans*, due to their abundance in the region and significant forensic value [33].

### Larval and newly emerged length and width measurement

The length of each larva was measured using a 150 mm plastic measuring vernier calliper (Hixon tools). Larvae were carefully placed on a flat, non-reflective surface to ensure accuracy. The calliper jaws were adjusted to span the length of the larva body, from the anterior end to the posterior end [41]. Similar to the body length, body width of each insect larva was measured using a digital calliper. Measurements were taken with minimal handling to avoid deformation or damage to the specimen [42]. Newly emerged insects were also measured using the same methods [42].

### Larvae and newly emerged insects weight measurement

The weight of larvae and newly emerged insect species were determined using a Benchmark Analytical Graphical Display DX Series Balance (Sigma-Aldrich). Larvae and newly emerged samples were individually placed on a weighing boat positioned on a weighing balance, and their weights were recorded to the nearest milligram [25].

### DNA extraction and PCR analysis

#### DNA extraction

Insects which were morphologically identified and those which were not easily identifiable morphologically (i.e. first and second instars) were subjected to DNA extraction. DNA was extracted from the legs and wings of newly emerged adult flies, beetles and from the entire anterior segments of beetle and fly larvae, using the Zymo Quick-DNA Miniprep Plus Kit, as per manufacturer’s instructions. Extracted DNA was then stored in -20 °C for further PCR analysis.

### PCR, gel electrophoresis and sequencing analysis

DNA from insects were amplified using the universal COI forward primer (LCO1490) and reverse primer (HCO2198) targeting the 658–708 bp regions [33, 43]. Successful amplicons were sent for sanger sequencing at Inqaba Biotec Industries Pty Ltd (South Africa) and edited using the BioEdit program [44]. Basic local alignment search tool (BLAST) was used to determine species identity, by comparing the edited sequences against the available closest match in the national centre for biotechnology information (NCBI) database.

### Statistical analysis

Morphometric data were recorded on Microsoft Excel and analysed using IBM SPSS (Version 30.0.0.). To assess the effect of paracetamol dosage on larval length, width, and weight, a one-way Analysis of Variance (ANOVA) was performed. When significant differences were detected, a post-hoc Tukey test was used to identify which group means differed significantly from one another. Statistically significant differences were determined at a threshold of *p* < 0.05, whilst Pearson’s correlation coefficient was used to examine the relationship between environmental factors (temperature and humidity) and the morphometric data.

## Results

### Oviposition and abundance of *Ch. putoria*, *Ch. megacephala*, *Ch. albiceps*, *L. sericata* and *T. micans* at different paracetamol doses

Insect colonization began on Day 1 for all carcasses; however, oviposition was delayed by 24 h in the paracetamol-treated carcasses. The first egg deposition occurred on Day 3 in the control carcass, whereas the treated carcasses exhibited oviposition only on Day 4. Initial oviposition by all dipteran species occurred primarily at the natural orifices such as the mouth, nostrils, ears, and eyes, followed sequentially by secondary sites such as the legs and abdomen for both intoxicated and control carcasses.

Overall, the total number of insects varied across the groups, with the control group showing a higher abundance (*n* = 228) compared to the paracetamol-treated carcasses: toxic *(n* = 165), lethal *(n* = 176), and double lethal (*n* = 163). However, the same species were observed in all the carcasses (Table 1). All dipteran species in paracetamol-injected carcasses showed reduced abundance in newly emerged flies compared to the control, while no newly emerged coleopterans were observed (Table 1). *Chrysomya putoria* larvae (*n* = 57) and newly emerged flies (*n* = 46) were the least abundant species observed across all carcasses, whereas *Ch. albiceps* larvae (*n* = 112) and newly emerged (*n* = 82) showed the highest abundance among the species collected (Table 1). Paracetamol-injected carcasses had a lower number of *Ch. Putoria*, *Ch. albiceps*, *L. sericata*, and *T. micans* larval stages compared to those observed and recovered from the control (Table 1). However, *Ch. megacephala* larvae were more common and abundant in carcasses treated with different doses of paracetamol (toxic, *n* = 25), (lethal, *n* = 20) and (double lethal, *n* = 23), compared to the control carcass (*n* = 18).

**Table 1:** Abundance of thirdinstar larvae and newly emerged adults of five dipteran species collected from pig carcasses intoxicated with different doses of paracetamol and control carcasses

Species collected	Developmental stage	Sample size (*n*)			
		Control	Toxic	Lethal	Double Lethal
*Chrysomya putoria*	Larvae	16	13	13	15
*Chrysomya megacephala*	Larvae	18	25	20	23
*Chrysomya albiceps*	Larvae	39	19	35	19
*Lucilia sericata*	Larvae	23	19	16	14
*Thanatophilus micans*	Larvae	51	33	36	33
*Chrysomya putoria*	Newly emerged	14	12	10	10
*Chrysomya megacephala*	Newly emerged	24	12	12	15
*Chrysomya albiceps*	Newly emerged	25	20	19	18
*Lucilia sericata*	Newly emerged	18	12	15	16
Total sample size (N)		**228**	**165**	**176**	**163**

### Effects of paracetamol doses on morphometry of third instars and newly emerged flies and beetles

**Fig. 1:**
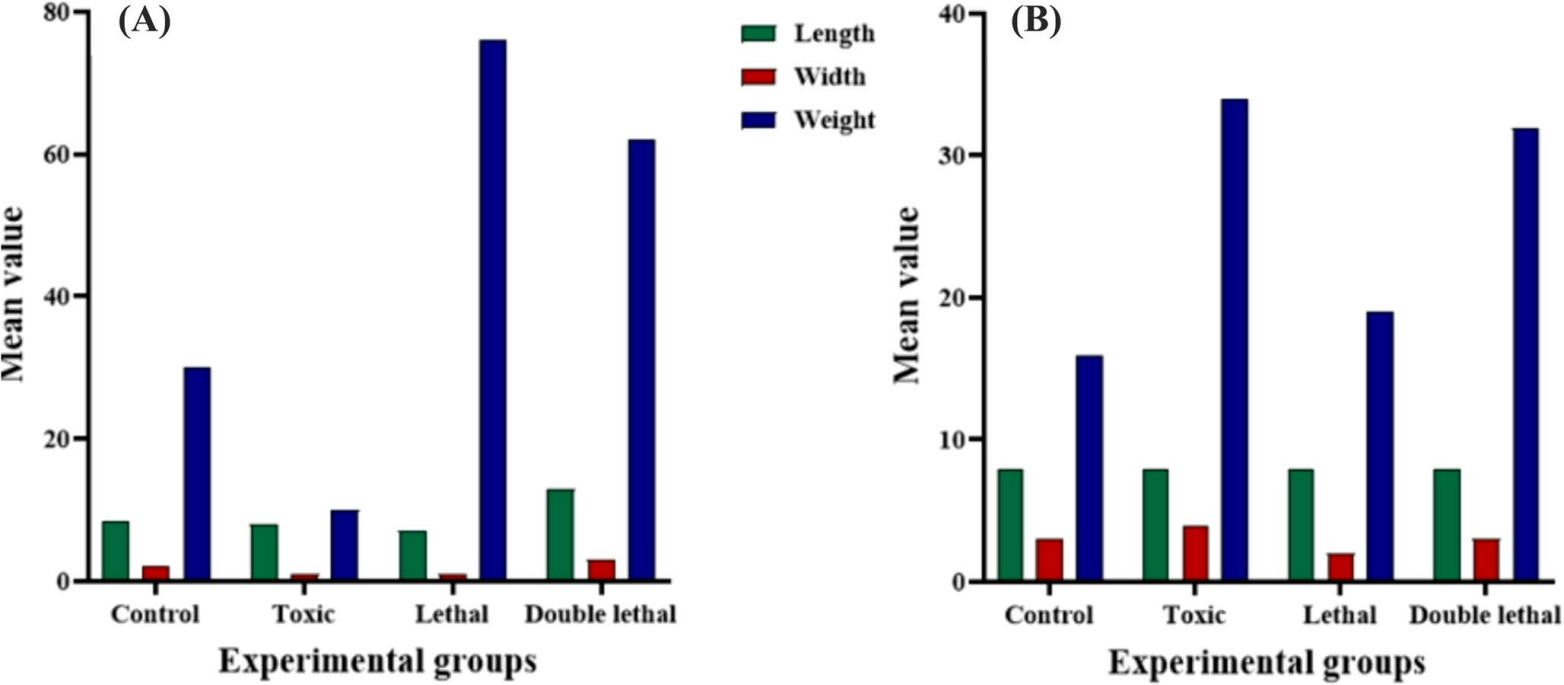
Morphometric measurements of mean length (mm), width (mm) and weight (mg) of *Chrysomia putoria* third-instar larvae (**A**) and newly emerged adults (**B**) for each experimental group (control, toxic, lethal, and double lethal)

**Fig. 2:**
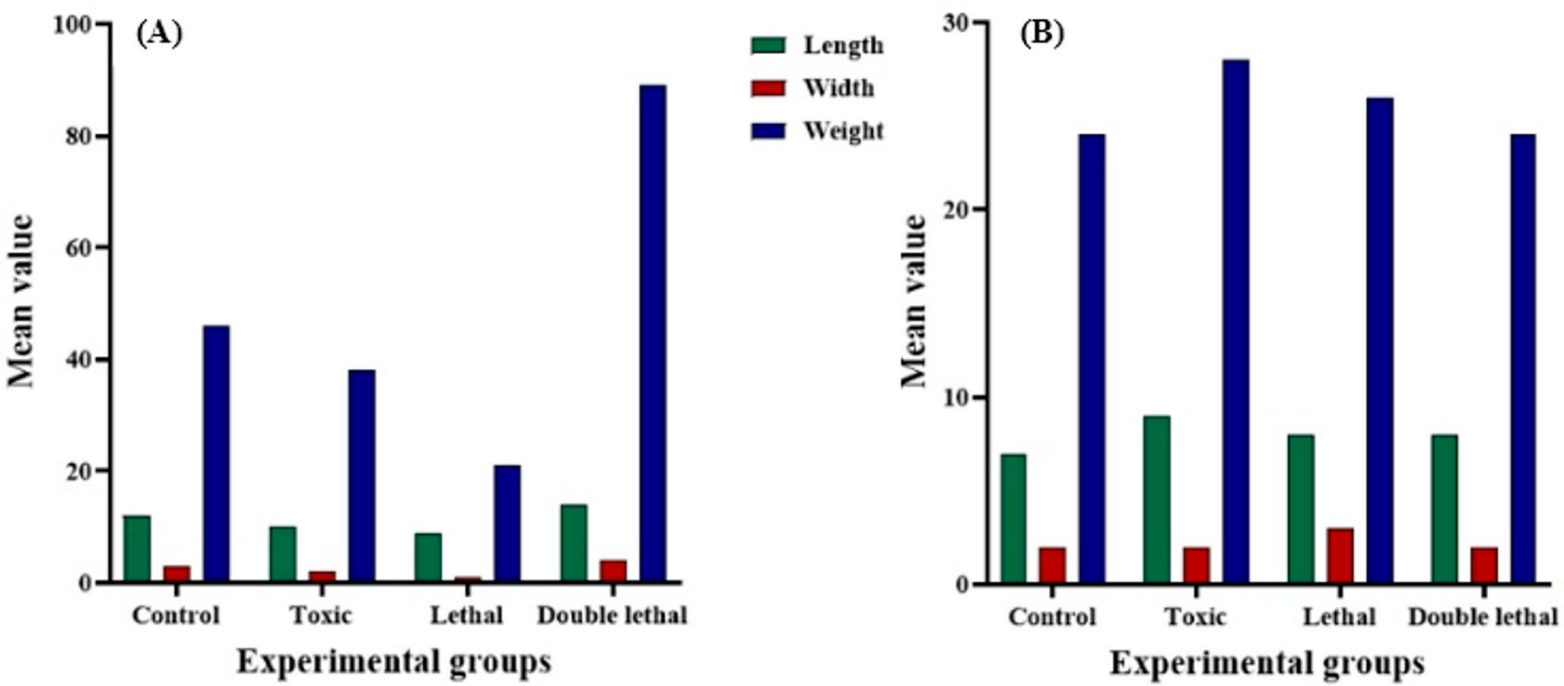
Morphometric measurements of mean length (mm), width (mm) and weight (mg) of *Chrysomia megacephala * third-instar larvae (**A**) and newly emerged adults (**B**) for each experimental group (control, toxic, lethal, and double lethal)

**Fig. 3:**
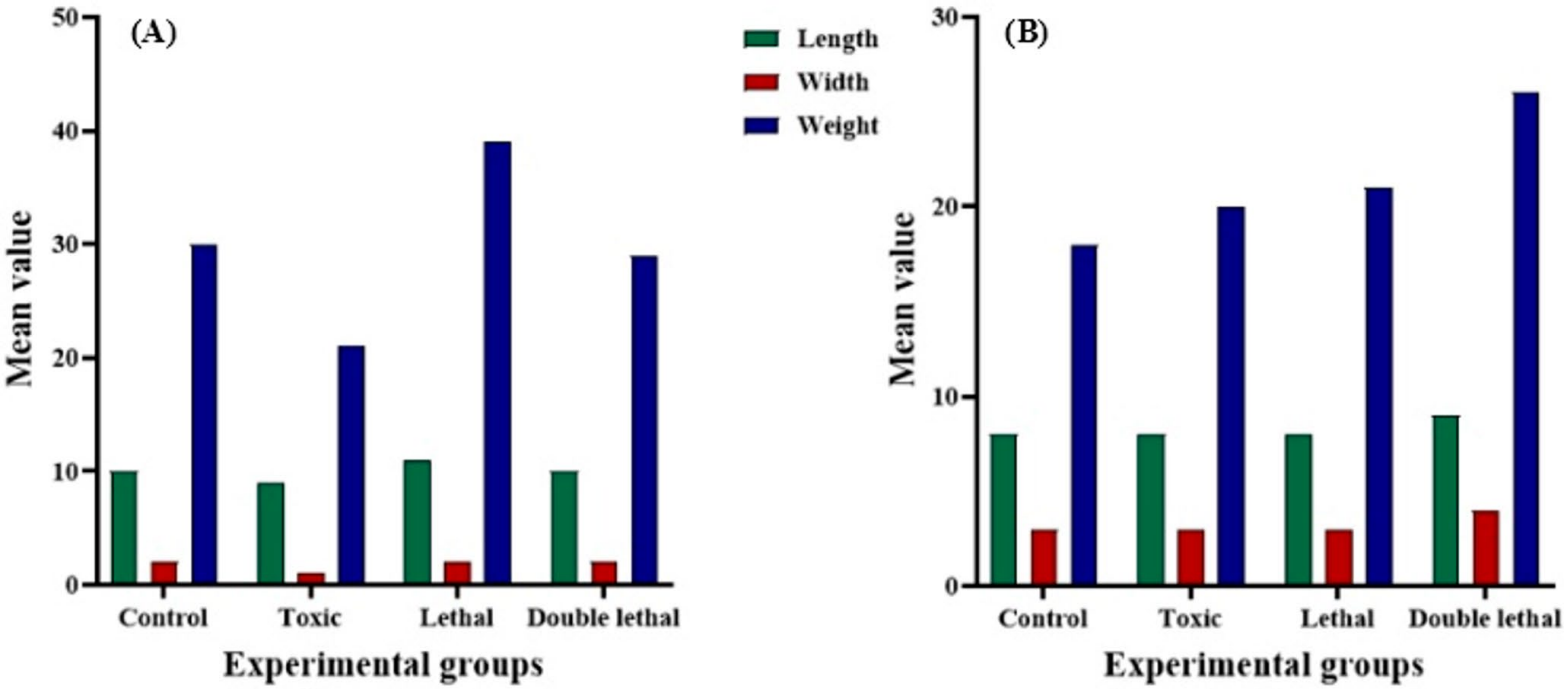
Morphometric measurements of mean length (mm), width (mm) and weight (mg) of *Chrysomia albiceps * third-instar larvae (**A**) and newly emerged adults (**B**) for each experimental group (control, toxic, lethal, and double lethal)

**Fig. 4:**
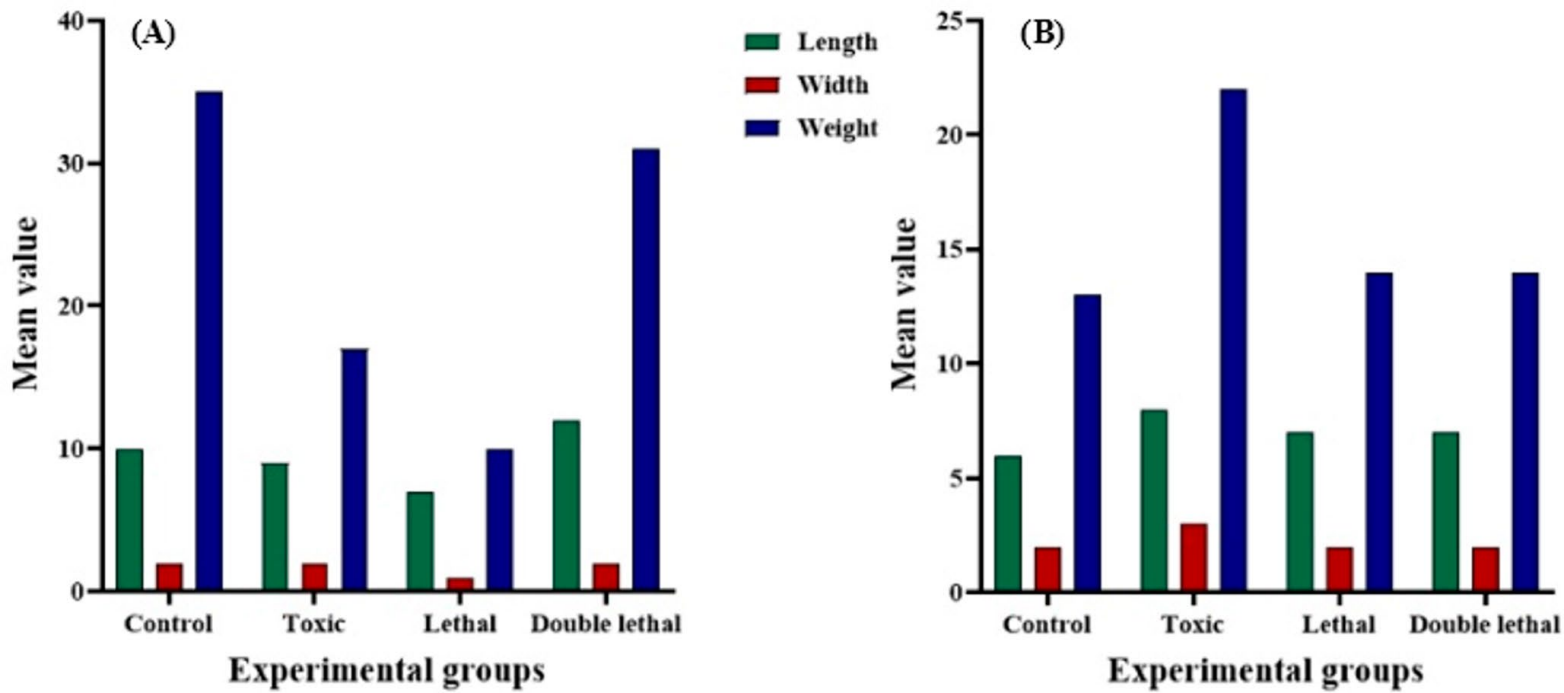
Morphometric measurements of mean length (mm), width (mm) and weight (mg) of *Lucilia sericata *third-instar larvae (**A**) and newly emerged adults (**B**) for each experimental group (control, toxic, lethal, and double lethal)

**Fig. 5:**
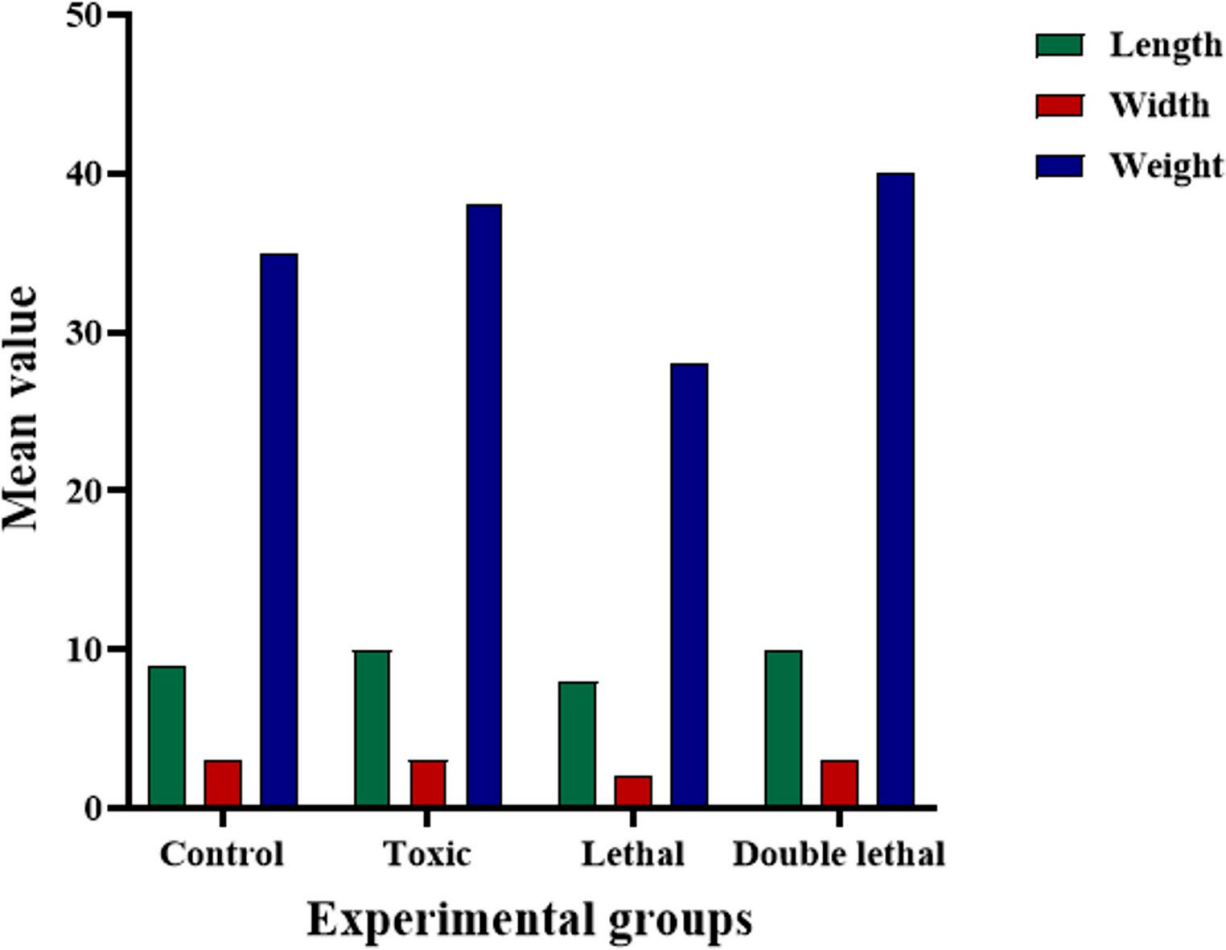
Morphometric measuremeaments of length (mm), width (mm) and weight (mg) of *Thanatophilus micans* larvae for each experimental group (control, toxic, lethal, and double lethal)

Results showed that the larval length of *Ch. putoria* (Fig. 1a) and *Ch. megacephala* (Fig. 2a) emerging from the double lethal dose had increased length compared to the control group. However, the toxic and lethal doses did not have any effects on the larval length of the same species (Fig. 1a) (Fig. 3a). Furthermore, larvae of *Ch. megacephala* (Fig. 2a) and *L. sericata* (Fig. 4a) exposed to lethal doses showed reduced length. Additionally, it was observed that all doses did not affect the larval length of *Ch. albiceps* (Fig. 3a), *T. micans* (Fig. 5) and the newly emerged adult flies (Figs. 1b, 2, 3 and 4b).

Results further showed that the double lethal dose of paracetamol increased the width of *Ch. putoria* and *Ch. megacephala* (Figs. 1a and 2a). However, all three doses of paracetamol had no effect on the larval width of *L. sericata*,* Ch. albiceps* and *T. micans.* Furthermore, the width of all four newly emerged adult dipteran flies were not affected by paracetamol. The weight of *Ch. putoria* (Fig. 1a) and *Ch. megacephala* (Fig. 2a) increased at the double lethal dose but *Ch. putoria* weight declined with the toxic and lethal doses. *Lucilia sericata* showed decreased weight across all three paracetamol dosages compared to the larvae collected from control carcass. Additionally, *Ch. albiceps* (Fig. 3) and *T. micans* (Fig. 5) demonstrated no effects on weight regardless of paracetamol dosage. Similarly, all paracetamol doses had no effects on the weight of all newly emerged adult dipteran species. A significant increase in length was observed in *Ch. putoria* species between the control and double lethal dose (*p* = 0.004) while *Ch. megacephala* weight decreased significantly (*p* = 0.046). *Lucilia sericata* showed significant effects at the lethal dose across all measured parameters length, width, and weight with p-values of 0.012, 0.001, and 0.001, respectively. No significant changes in length, width, or weight were detected for *T. micans* larvae and newly emerged adults of *Ch. putoria*,* Ch. megacephala*,* Ch. albiceps*,* and L. sericata*, across all paracetamol doses. A weak to no correlation (*r* = 0.32) was observed between ambient temperature and humidity and the morphometric measurements in both control and paracetamol-treated carcasses, suggesting minimal environmental impact on the observed differences.

### Effect of paracetamol doses on the morphometry of adult insects

**Fig. 6:**
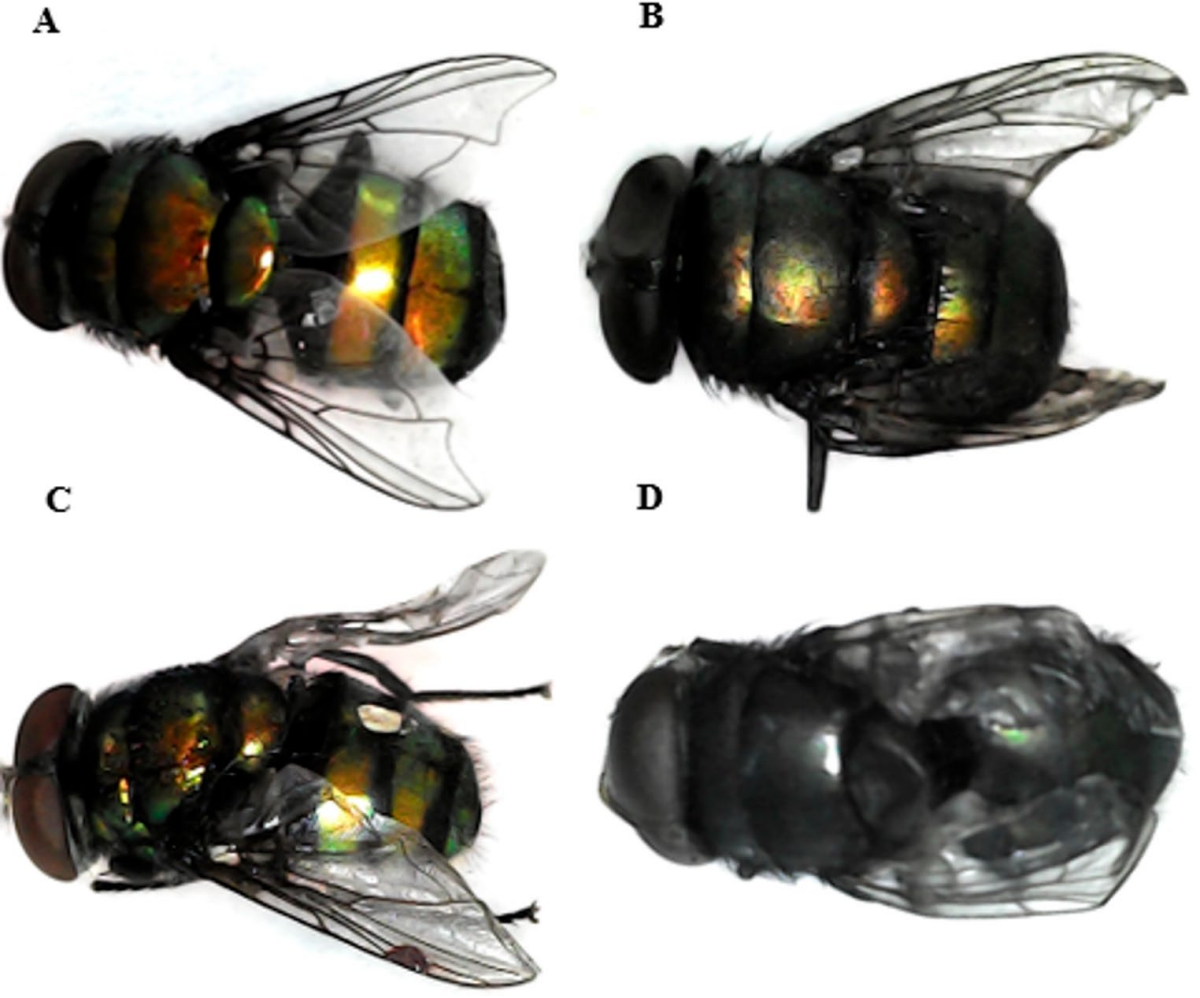
Newly emerged flies of *Chrysomia putoria* from (**A**) the control group showing no abnormalities, (**B**) from the toxic dose group with slight wing abnormality, (**C**) from the lethal dose group showing deformation in wings and slight loss of colour and (**D**) from the double lethal dose group showing severe deformation in wings and complete discoloration

**Fig. 7:**
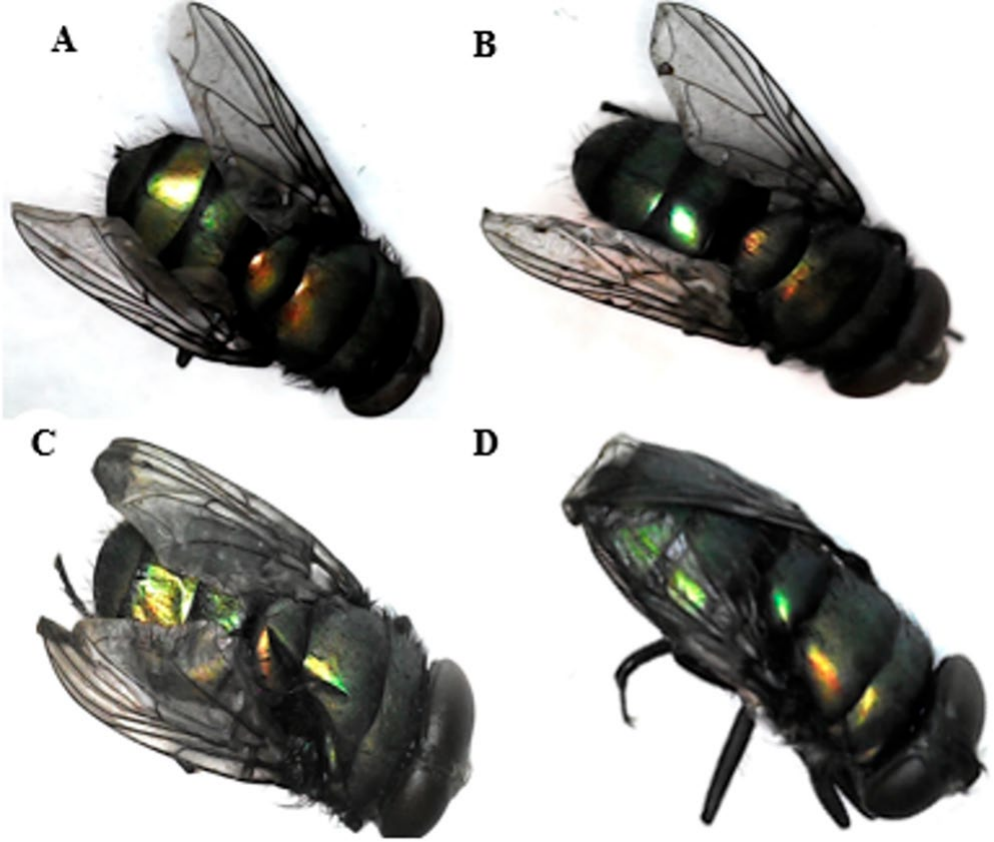
Newly emerged flies of *Chrysomia megacephala* with (**A**) no abnormalities, from the control group, (**B**) no abnormalities, from the toxic dose group, (**C**) showing deformation in wings from the lethal dose group, and (**D**) showing deformation in wings from the double lethal dose

**Fig. 8:**
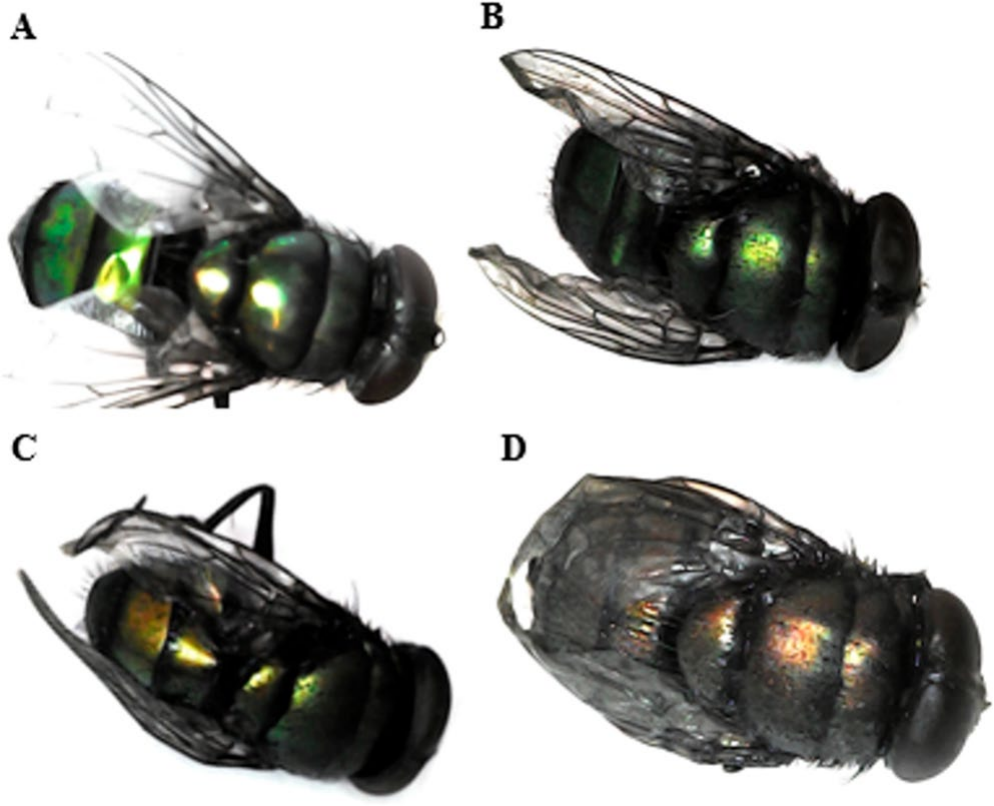
Newly emerged flies of *Lucilia sericata* showing (**A**) the normal control colony, with no abnormalities, (**B**) slight discoloration and wing abnormality from the toxic dose group, (**C**) showing deformation in wings and slight loss of colour from the lethal dose group and (**D**) showing severe deformation in wings and discoloration double lethal dose group

Insects collected from the clean carcass displayed no morphological abnormalities (Figs. 6, 7 and 8a), while *Ch. putoria* and *L. sericata* collected from all paracetamol-treated groups presented wing deformities at the toxic and the lethal doses, and severe wing deformities along with complete discoloration at the double lethal dose (Figs. 6b-d and 8b-d). Additionally, *Ch. megacephala* (Fig. 7b-d) displayed normal wing formation and coloration in the toxic groups, while the lethal and double lethal groups showed deformed wings and partial discoloration. Furthermore, *Ch. albiceps* showed no observable deformities and discoloration across the control and all treated carcasses. Overall, proportional abnormalities in all dipteran species were lower in the control group whereas, the double lethal dose group exhibited the highest proportion of abnormalities compared to the control, toxic, and lethal groups. Among the observed abnormalities, discoloration was the most prevalent, exceeding the proportion of wing deformities (Table 2).

**Table 2:** Proportions of morphological abnormalities (discoloration and wing deformities) observed in dipteran species across all experimental groups

Species collected	Morphological abnormality	Proportion affected			
		Control	Toxic	Lethal	Double lethal
*Chrysomya putoria*	Discoloration Deformed wings	10% (1/14) 0% (0/14)	40% (5/12) 10% (1/12)	70% (7/10) 40% (4/10)	90% (9/10) 60% (6/10)
*Chrysomya megacephala*	Discoloration Deformed wings	10% (2/24) 5% (1/24)	10% (2/12) 20% (2/12)	70% (10/12) 50% (6/12)	80% (12/15) 70% (10/15)
*Chrysomya albiceps*	Discoloration Deformed wings	0% (0/24) 0% (0/24)	0% (0/12) 0% (0/12)	0% (0/12) 0% (0/12)	0% (0/12) 0% (0/12)
*Lucilia sericata*	Discoloration Deformed wings	0% (0/18) 0% (0/18)	10% (2/12) 20% (2/12)	50% (9/18) 25% (4/18)	80% (13/16) 50% (8/16)

### Effect of paracetamol on the mortality of insects

The survival rate of larvae was 100% in the control carcass and all treated carcasses regardless of paracetamol dosage. However, mortality was observed in newly emerged adult flies with a significant variation between paracetamol dosage groups. The mortality of newly emerged *Ch. putoria*, *Ch. megacephala*, *Ch. albiceps* and *L. sericata* increased with the paracetamol dose. At a toxic dose, the mortality was 22.2%, 35.0% at a lethal dose and 45.0% at a double lethal dose. Nevertheless, in the control group the mortality rate for the same species was 11.1%, respectively.

## Discussion

Several studies have demonstrated that the presence of toxicants and drugs in a decomposing body can interfere with the attraction, oviposition, and development of carrion-feeding insects [12, 45–47]. In this study, the presence of paracetamol in decomposing pig carcasses impacted the number of *Ch. putoria*,* Ch. albiceps*, *L. sericata* and *T. micans* attracted to the intoxicated carcasses as more of these species were abundant in the control compared to the intoxicated carcasses. This variation in abundance may be due to the differences in odor profiles released by paracetamol treated and control carcasses, corresponding with previous studies that unspiked (control) and drug treated carcass often release different odours which can either delay or accelerate the attraction of insects to the carcass [48, 49].

Mahat et al. [50] and Abo El-Ela et al. [46] reported that carcasses treated with malathion and clozapine respectively, displayed delayed oviposition compared to the control carcasses. Similar observations were made in this study where oviposition was delayed by 24 h in paracetamol treated carcasses, potentially caused by the repellent effects of drugs on certain insect species which resulted in delayed oviposition. Furthermore, while certain toxins and drugs can influence insect oviposition site selection, this was not observed in the current study as the initial oviposition occurred in the body orifices (mouth, ears, eyes, and nostrils) in all carcasses. This pattern aligns with observations from other studies where toxic substances did not influence the oviposition preferences [47, 51].

The length, weight and/or width of larvae are essential parameters used to measure the rate of development, and these are crucial in determining PMI. However, Bhardwaj et al. [6] also highlighted that type and concentration of these toxins or drugs can affect these parameters differently in various arthropods species [3]. The present study showed that various doses of paracetamol had different effects on the larval length of studied insect species, which indicated a dose dependent relationship and variation between species. For instance, *Ch. putoria* and *Ch. megacephala* larvae from the double lethal dose of paracetamol had increased length, whilst larval length of larva of *Ch. putoria* and *Ch. albiceps* from the toxic and lethal doses did not differ from the control. Several studies also highlighted the same inconsistencies as observed in the current study, where Souza et al. [52] observed an increase in the larval length of *Ch. megacephala* and *Ch. putoria* exposed to nandrolone, while Galil et al. [53] and Rezende et al. [37], reported reduced larval length of *Ch. megacephala* exposed to dimethoate and *Ch. putoria* treated with phenobarbital respectively. However, paracetamol had no effect on the larval length of *Ch. albiceps* and *T. micans*, and these may be associated with differences in feeding patterns and digestive systems between different species. These findings are consistent with independent studies by Fathy et al. [54] and Keshavarzi et al. [55] who both reported no effects of codeine phosphate and methadone on larval development of *Ch. albiceps* and *Calliphora. vicina* respectively.

Overall paracetamol’s effects on developmental parameters varied across species, the double lethal dose increased the larval width and weight of *Ch. putoria* and *Ch. megacephala*, while toxic and lethal doses reduced the larval weight *Ch. putoria* and all three doses reduced the larval weight of *L. sericata*. In contrast, no significant effects were observed on the larval width or weight of *Ch. albiceps*, *T. micans*, and all newly emerged adult flies, indicating that drug effects on developmental parameters differ with species and should be evaluated independently.

Morphometric parameters can be influenced by environmental factors such as temperature and humidity [56, 57]. However, a weak to no correlation was observed between ambient temperature and humidity and the differing morphometric data in both control and treated paracetamol carcasses in this study. Thus, suggesting that the environmental factors had minimal impact on the observed differences. Our results further showed that no morphological abnormalities or deformities were observed in all larvae collected from intoxicated carcasses, suggesting that paracetamol did not interfere with the external structural development of larvae as observed by O’Brien and Turner [25]. However, newly emerged flies of *Ch. putoria*, *Ch. megacephala* and *L. sericata* displayed varying levels of abnormalities on their wings, which were either discolored and not fully expanded compared to similar species emerged from the control carcass. These abnormalities observed across species, could be attributed to paracetamol interfering with biochemical and metamorphic pathways essential for proper cuticle hardening, pigmentation, and wing expansion [46]. Similar abnormalities were described by Abo El-Ela et al. [46] as well as Gaur and Kumar [58], where medicinal plant and clozapine caused the wings of newly emerged *Sarcophaga ruficornis* to be deformed and unable to inflate and stretch fully respectively. Furthermore, these abnormalities varied with the species and dose concentration, highlighted by a more prominent and pronounced discoloration in *Ch. putoria* at a higher dose compared to other doses and other species (*L. sericata* and *Ch. megacephala*). Moreover, *Ch. megacephala* showed slight discoloration only at lethal and double lethal doses compared to the lower dose, supporting previous studies where toxic effects on morphology were apparent at a higher drug concentrations compared to the lower doses [3, 59–61]. The results further revealed that all doses of paracetamol did not have any effect on the morphology of *Ch. albiceps* newly emerged flies, indicating that drugs may have different outcomes on different species studied [6]. Therefore, the effects of a specific drug on different dipteran fly species may not be generalized as they vary with different species.

There were no recorded mortalities in larvae from all dipteran and coleopteran species, in all pig carcasses. These findings contradicted with Galil et al. [53] who reported increased mortality in larvae exposed to a higher dosage of dimethoate. Abo El-Ela et al. [46] and Afifi et al. [62] also recorded an increased mortality in *S. ruficornis* and *Sarcophaga argyrostoma* pupae collected from the highest dose of clozapine and clonazepam respectively compared to lower doses. These results contradict earlier observations by Goff et al. [11], who reported a lower larval mortality rate of *L. sericata* at a higher dose of methylenedioxymethamphetamine reared from the rabbit carcasses. However, this study noted increased mortality with higher drug dosage in newly emerged *Ch. putoria*, *Ch. megacephala*, *Ch. albiceps* and *L. sericata* flies, with the highest mortality recorded at the double lethal dose of paracetamol. These inconsistencies in the results supports previous studies that different drugs and concentrations may have different and/or similar effects on different species [6, 9, 11, 23, 40].

Overall, the differential response observed between species implies that the effects of paracetamol on insect development are highly species-specific, with some species exhibiting more resilience or less sensitivity to the toxic effects of the drug [63]. To avoid inaccurate PMI estimations and cause of death, the effect of different drugs must be considered during forensic investigation involving drug related cases where entomological data is used as evidence [64]. This study contributes to forensic entomotoxicology by providing empirical data on the impact of a commonly used over-the-counter drug, paracetamol, on insect development, expanding existing entomotoxicology reference databases of commonly abused drugs.

## Conclusion

The study findings revealed that paracetamol significantly influenced the attraction, oviposition patterns, and developmental parameters of carrion-feeding insects, with notable variations in species-specific responses with the exception of *Ch. megacephala*, which was more abundant on a carcass spiked with a higher dose of paracetamol. These results further indicate that dipteran species feeding on high doses of paracetamol can have different effects which in turn can affect PMI estimations in forensic cases involving paracetamol overdose. The findings contribute new knowledge on the effects of paracetamol on the development and morphometry of selected forensically important arthropod species in the KwaZulu-Natal Province of South Africa. Subsequently the results will enhance the accuracy of PMI estimation in forensic investigations, particularly in suspected homicide and suicide cases involving paracetamol in this region. The authors recommend further studies to determine the effects of diverse drugs on the development of different carrion feeding insects since the effect of different drugs vary with the drug type and insect species. 
